# A truncated anti-CRISPR protein prevents spacer acquisition but not interference

**DOI:** 10.1038/s41467-022-30310-x

**Published:** 2022-05-19

**Authors:** Cécile Philippe, Carlee Morency, Pier-Luc Plante, Edwige Zufferey, Rodrigo Achigar, Denise M. Tremblay, Geneviève M. Rousseau, Adeline Goulet, Sylvain Moineau

**Affiliations:** 1 Département de biochimie, microbiologie, et bio-informatique, Faculté des sciences et de génie, Université Laval, Québec City, QC Canada; 2 Groupe de recherche en écologie buccale, Faculté de médecine dentaire, Université Laval, Québec City, QC Canada; 3 Département de médecine moléculaire, Faculté de médecine, Université Laval, Québec City, QC Canada; 4 Laboratorio de Microbiología Molecular, Departamento de Biociencias, Facultad de Química, Universidad de la República, Montevideo, Uruguay; 5 Félix d’Hérelle Reference Center for Bacterial Viruses, Faculté de médecine dentaire, Université Laval, Québec City, QC Canada; 6 Laboratoire d’Ingénierie des Systèmes Macromoléculaires, Institut de Microbiologie, Bioénergies et Biotechnologie, CNRS UMR7255, Aix-Marseille Université, Marseille, 13402 France

**Keywords:** Phage biology, Bacteriophages, Bacteria, Virus-host interactions

## Abstract

CRISPR-Cas systems in prokaryotic cells provide an adaptive immunity against invading nucleic acids. For example, phage infection leads to addition of new immunity (spacer acquisition) and DNA cleavage (interference) in the bacterial model species *Streptococcus thermophilus*, which primarily relies on Cas9-containing CRISPR-Cas systems. Phages can counteract this defense system through mutations in the targeted protospacers or by encoding anti-CRISPR proteins (ACRs) that block Cas9 interference activity. Here, we show that *S. thermophilus* can block ACR-containing phages when the CRISPR immunity specifically targets the *acr* gene. This in turn selects for phage mutants carrying a deletion within the *acr* gene. Remarkably, a truncated *acrIIA* allele, found in a wild-type virulent streptococcal phage, does not block the interference activity of Cas9 but still prevents the acquisition of new immunities, thereby providing an example of an ACR specifically inhibiting spacer acquisition.

## Abstract (web summary)

Phages can use ACR proteins that inhibit the adaptive immunity activities of bacterial CRISPR-Cas systems. Here, Philippe et al. show that these systems can block ACR-containing phages by targeting the *acr* gene, and this can select for phage mutants carrying a deletion within *acr* that does not block DNA cleavage (interference) but prevents the addition of new immunity (spacer acquisition).

## Introduction

Bacteriophages prey on bacteria, often leading to cell lysis. To defend against a genetically diverse phage population, bacteria have developed an extensive arsenal of defense mechanisms^1–4^. In parallel, phages have adapted a myriad of strategies to bypass these anti-phage hurdles^5^. This biological arms race is particularly well illustrated with the CRISPR-Cas system. CRISPR (Clustered Regularly Interspaced Short Palindromic Repeats)-Cas (CRISPR associated proteins) is a system that has an adaptive immunity memory that recognizes and then cleaves invading foreign nucleic acids. Its mode of action usually relies on three general steps: (1) adaptation step, in which a short nucleotide sequence (spacer) mostly from invading DNA, is integrated between two repeats within the CRISPR array, (2) RNA biogenesis step, in which the CRISPR array is transcribed and matured into short crRNAs, (3) interference step, in which the Cas nuclease complex armed with a crRNA binds to a matching DNA or RNA and cleaves it, resulting in sequence specific immunity.

*Streptococcus thermophilus* is a Gram-positive bacterium found in milk-related environments and is used by the dairy industry to manufacture yogurt and speciality cheeses^6^. For decades, the dairy industry has been closely monitoring phage diversity as well as implementing various strategies to limit the propagation of virulent phages in industrial settings in order to complete successful milk fermentation processes^7^. Of interest, this streptococcal species primarily relies on Cas9-containing CRISPR-Cas systems (type II-A) to defend against virulent phages. While most cells from a phage-sensitive bacterial population will rapidly die when infected by virulent phages, a few phage-resistant cells will also survive. These *S. thermophilus* derivatives, called Bacteriophage-Insensitive Mutants (BIMs), are usually resistant to phages due to the integration into the CRISPR array of a new spacer captured from the invading phage genome. The presence of active CRISPR-Cas systems also offers the opportunity to change the immunity profile of a given strain by naturally adapting its CRISPR profile to include spacers that target conserved genes in phages present in a particular ecosystem. Thus, this natural mechanism can lead to the development of bacterial strains that are resistant to a wide variety of phages. However, the recent discovery of phages carrying ACR proteins that block Cas9 interference activity, is now limiting its usefulness^8,9^. Two families of functional ACR proteins, AcrIIA5 and AcrIIA6, have been found in *S. thermophilus* phages^10,11^.

Here we show how bacteria can evade ACR phages by using CRISPR-immune strains that specifically target the *acr* genes. In our experiments, we observed phage-resistant bacterial phenotypes that exploited CRISPR-Cas with a very specific *acr* target. Phage-resistant mutants were found to have deletions in their *acr* gene in the target location. We also observed that a newly-isolated phage that infects *S. thermophilus* harbored a truncated allele of AcrIIA6 that did not block CRISPR interference, but considerably reduced spacer acquisition rate. By showing that the function of this truncated allele is to impede spacer acquisition, we were able to test and confirm the existence of anti-adaptation ACRs.

## Results and discussion

In an attempt to find a strategy to inhibit ACR+ virulent phages, we considered that perhaps a spacer targeting an *acr* gene could short-circuit the viral production of ACRs and provide a CRISPR-based phage resistance phenotype. To test our hypothesis, we used the model wild-type strain *S. thermophilus* DGCC7710, which contains two active type II-A CRISPR-Cas systems (CR1 and CR3) and is sensitive to several phages, including the three virulent phages, 2972 (contains no ACR), D1126 (contains AcrIIA5), and D3288 (AcrIIA6). We then generated two BIMs, each carrying a different spacer^12^, one spacer targeting *acrIIA5* (BIM-D1126_ACR_) and the other targeting *acrIIA6* (BIM-D3288_ACR_). We also used a previously described BIM (SMQ-1335b)^12^, carrying a spacer targeting an early-expressed gene encoding for the primase, a conserved region in the three phage genomes (BIM 3 phages).

**Fig. 1:**
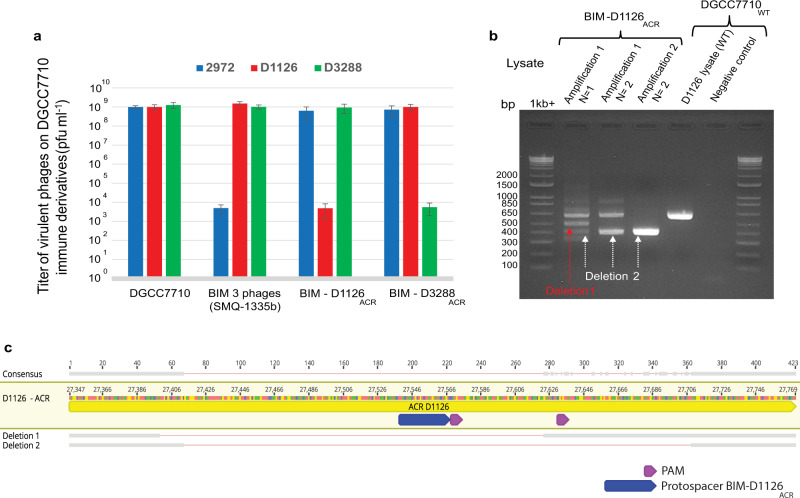
A spacer that targets the *acr* gene provides phage resistance and leads to selection of phage-escaping mutants (CEM) containing a deleted *acr* gene. **a** Titers of phages carrying no ACR (phage 2972 in blue) or AcrIIA5 (D1126 in red) or AcrIIA6 (D3288 in green), on four *S. thermophilus* strains, namely the phage-sensitive strain DGCC7710, strain SMQ-1335-b containing a spacer in its CR1 array perfectly matching a conserved region in the three phage genomes, strain BIM-D1126_ACR_ containing a spacer matching the *acrIIA5* gene of phage D1126, and strain BIM-D3288_ACR_ containing a spacer matching the *acrIIA6* gene of phage D3288. The BIMs carrying a spacer targeting an *acr* gene provided a significant phage resistance phenotype (lower phage titers). ﻿The titers are from three biological replicates and each of two technical replicates. Error bars represent standard deviation. **b** PCR analyses on phage lysates based on the amplification of phage D1126 infecting the BIM that targets its *acr* (BIM-D1126_ACR_). The *acr* gene was amplified by PCR after two rounds of phage amplification. The gel shows deleted (lanes 2–4) and full-length (lane 5) PCR products for the *acr* in the CEM and wild-type phage D1126, respectively. **c** Nucleotide sequence alignment of the deleted and wild-type *acr* genes (PCR products) shows deletions within the targeted protospacer region.

As expected, the bacterial strain *S. thermophilus* SMQ-1335b was resistant to phage 2972 (5 log reduction in phage titer) but was sensitive to the two ACR+ phages, D1126 and D3288 (Fig. 1a). Interestingly, both BIMs carrying a spacer targeting an *acr* gene were resistant (5 log reduction) to their respective ACR+ phage (Fig. 1a). These data clearly indicate that spacers targeting an *acr* gene offers a significant mean to combat ACR+ phages.

Then, we examined if we could isolate CRISPR-escaping phage mutants (CEM) as reported previously with other BIMs^13^. As expected, CEMs with single nucleotide mutation in the targeted protospacer regions (or in their protospacer-adjacent motifs or PAM) were identified in 20/20 plaques recovered (at EOP of 10^−5^) from the infection of strain SMQ-1335b with phage 2972. However, no mutations were found in the *acr* gene of 50 isolated plaques (EOP 10^−5^) from the infection of BIM-D1126_ACR_ with phage D1126 or from 50 plaques from the infection of BIM-D3288_ACR_ with D3288. These data suggest that some ACR+ phages are likely capable of producing some anti-CRISPR proteins in these *acr*-targeting BIMs for the benefit of other infecting phages as observed by others^14,15^. Indeed, at sufficient density, it has been previously shown that ACR+ phages are able to cooperate to overcome CRISPR immunity, with a first phage genome being cleaved by Cas9 but apparently after producing enough ACR proteins within the cell for the benefit of a second infecting wild-type ACR+ phage. It should be noted that *acr* genes in streptococcal phage genomes are located in the replication module, which is expressed early in the phage infection process^16^.

We also tried to detect CEMs after infecting *acr*-targeting BIMs with its respective ACR+ phage in liquid medium, allowing phage infections on planktonic, non-structured culture. We performed a PCR analysis on the phage lysates by amplifying the phage *acr* gene. With phage D1126 harboring AcrIIA5, we observed deletions in the *acr* gene after a few phage replications in liquid medium (Fig. 1b). Sanger sequencing of the PCR products revealed two deletions both spanning the targeted protospacer and its PAM within the *acr* gene (Fig. 1c, Supplementary Fig. S12). Additional phage replications in liquid medium led to a unique PCR product containing only the larger deletion (Fig. 1b, c). We were able to isolate these phage mutants with a deleted *acr* gene, and they could no longer replicate on SMQ-1335b, indicating that the truncated ACR could no longer block Cas9 interference activity. Surprisingly, we could not observe deletions in similar experiments in liquid medium with phage D3288 carrying AcrIIA6 and its *acr*-targeting BIM. Nonetheless, these data showed that virulent phages can adapt to *acr*-targeting spacer by deleting part of their *acr* gene but with the cost of no longer being able to block Cas9 immunity.

These data also suggested that truncated viral ACRs may be remnants of the arms race between phages and bacteria. To support this, we searched for the presence of other truncated *orfs* among non-essential genes in the replication module of virulent *S. thermophilus* phages. Specifically, we aligned homologs of ORF27_2972_ and ORF31_DT1_ (Supplementary Fig. S1) and their upstream region and found evidence of mutations and deletions leading to truncation at the N-terminus, modifying the start codon. These data indicate that other phage proteins can also be truncated. Interestingly, we did not find spacers matching these truncated regions in *orf27* and *orf31*. We then searched databases for the presence of other similarly truncated *acrIIA* (see the “Methods” section). Truncations were observed with at least six other AcrIIAs from other bacterial genera or species, and still likely forming ORFs in plasmids or (pro)phages genomes (Supplementary Figs. S2 to S7).

**Fig. 2:**
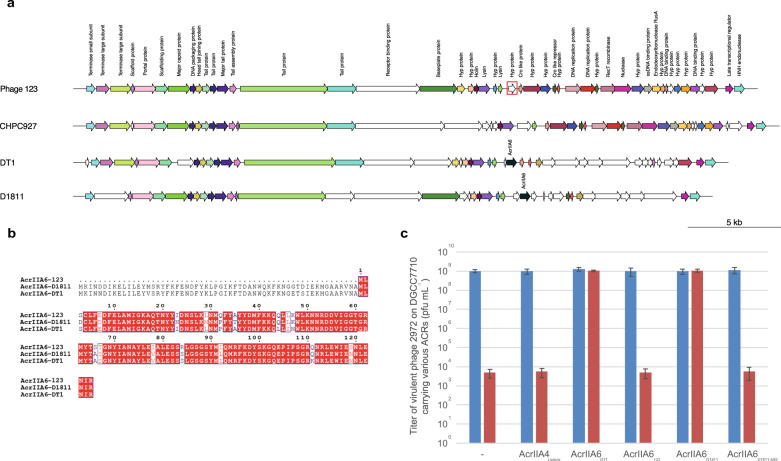
The truncated AcrIIA6 of phage 123 inhibits spacer acquisition but not CRISPR interference. **a** Genome architecture of phage 123 and its relatedness to a few other *S. thermophilus* phages. ORFs that share over 80% amino acid identity are colored. The most closely related phage is CHPC927. The position of the truncated *acrIIA6* gene on the phage 123 genome is indicated by a red square. **b** Sequence alignment of AcrIIA6 variants reveal a deletion of the first 59 amino acid residues in AcrIIA6_123_ as compared to previously characterized full-length AcrIIA6 from other phages. **c** Titer of phage 2972 on its host strain *S. thermophilus* DGCC7710 or on a CR1-immune derivative (SMQ-1335b), carrying either the empty vector pNZ123 or the vector expressing AcrIIA4 (inactive in *S. thermophilus*) or functional AcrIIA6 alleles. Resistance to phage 2972 by CRISPR interference was observed in the control (empty vector), with AcrIIA4, with AcrIIA6_123_ and with a truncated version of AcrIIA6_D1811_ (AcrIIA6_D1811-Δ59_). Each column shows the average of three biological replicates and each of two technical replicates.

We also found in our streptococcal phage collection, the virulent wild-type phage 123, which naturally harbors a truncated *acrIIA6* allele. Phage 123 is closely related to the reference virulent streptococcal phage DT1, which possesses a full-length AcrIIA6 (Fig. 2a). The truncated *acr* in phage 123 is positioned in the same genomic location as other previously described *acr* genes in *S. thermophilus* phages^10,11^. Protein sequence alignment revealed that the truncated AcrIIA6 of phage 123 is missing the first 59 amino acids of typical AcrIIA6 proteins (Fig. 2b, Supplementary Fig. S1 and S8). We used the host strain UY04 and challenged it with phage 123. New spacers were acquired in the tested BIMs but only in CR3. No spacer acquisition was detected in CR1 (Supplementary Fig. S9), similar to what had been previously observed when a functional ACR was present^10,11^. To assess if AcrIIA6_123_ blocks the interference activity of Cas9, its gene was cloned into an expressing vector (pNZ123), which was then transformed into the model strain DGCC7710 and a CR1-immune derivative (SMQ-1335b) to perform an interference assay. Our results revealed that the short ACR of phage 123 variant did not block CRISPR interference and thus appeared non-functional (Fig. 2c), as observed with the different deleted ACR versions generated in liquid medium (Fig. 1b, c).

**Fig. 3:**
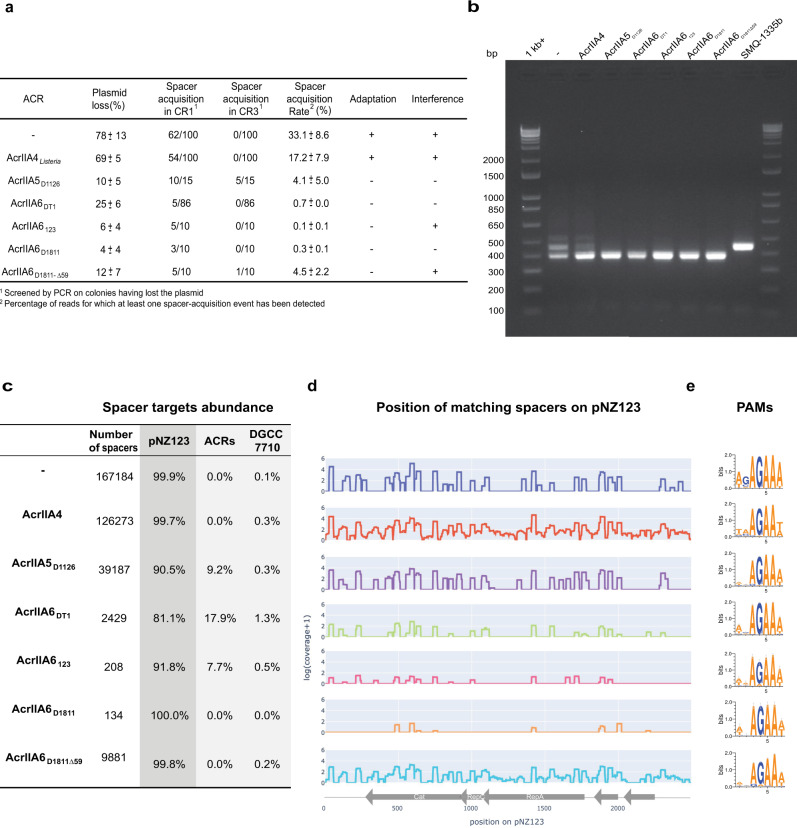
Acquired spacers primarily target the plasmid sequence with no preference for a specific location. **a** The presence of AcrIIA6 variants provides plasmid stability by inhibiting plasmid lost due to the type II-A CRISPR-Cas system. Spacer acquisition was evaluated by PCR on colonies that had lost the plasmid and by Illumina sequencing of the CR1 array at the end of the experiment, from two biological replicates. Truncated AcrIIA6 alleles maintained the plasmid and only a low level of spacer acquisition was detected. **b** PCR amplification of CR1 prior to Illumina sequencing on *S. thermophilus* cultures after 9 passages in the plasmid loss assay. **c** Relative abundance of spacers perfectly matching the plasmid, the *acr* gene, or the bacterial genome. **d** Spacers targeting the plasmid pNZ123 were mapped but no patterns were detected for target locations. **e** Seven nucleotides were extracted upstream from each spacer that targeted the plasmid sequence to identify the cognate PAMs for each condition and are represented using WebLogo. These results are from one biological replicate, the second replicate is shown in Figure S11.

However, the presence and to some extent the maintaining of a truncated *acr* gene in the genome of a virulent phage, which is otherwise usually rather compact, was intriguing. Thus, we envisioned that truncated ACR may still block CRISPR immunity but perhaps differently. As mentioned previously, the first step of the CRISPR-Cas system is the adaptation, in which a new spacer is integrated into the CRISPR array^17^. However, it is not always trivial to differentiate the adaptation activity from interference activity as they are often intertwined in phage assays. To circumvent this issue, we performed a plasmid loss assay as previously described^18,19^. This assay involved the serial propagation of the *S. thermophilus* strain DGCC7710, carrying a plasmid with an antibiotic-resistance marker, in liquid medium without antibiotic pressure. Previous studies have shown that under these growing conditions, the plasmid pNZ123 is not stable in this strain (after 60 generations), due to plasmid-derived spacer acquisition and subsequent interference activity of the CRISPR-Cas system in this strain^12,18^. We confirmed these previous data since here as 78% of the colonies tested lost their ability to grow on chloramphenicol, which was due to the acquisition of plasmid-targeting spacers in the CR1 array (Fig. 3a).

In parallel, we selected representatives of known efficient ACR (blocking interference) from various streptococcal phages: AcrIIA5_D1126_, AcrIIA6_D1811_, and AcrIIA6_DT1_ and which were cloned separately into pNZ123 (Fig. 2c). We also cloned the truncated AcrIIA6_123_ as well as a shorter version of AcrIIA6_D1811_, in which the first 59 amino acids were removed (AcrIIA6_D1811-Δ59_). The AcrIIA4 from a *Listeria* prophage, previously shown to be inactive in *S. thermophilus*, was also used as a negative control. Plasmid loss assays showed that pNZ123 carrying AcrIIA5 was significantly more stable than pNZ123 alone in *S. thermophilus*, with only 10% of plasmid loss (Fig. 3a). Similar results were obtained with plasmids carrying full-length AcrIIA6 or truncated versions (Fig. 3a). Among the AcrIIA6 variants tested, AcrIIA6_D1811_ was the most efficient in maintaining plasmid stability while AcrIIA6_DT1_ was the least efficient, even though these two variants share 92% amino-acids identity. These data suggest that mutations in key amino acids may alter their activities. As expected, pNZ123 carrying AcrIIA4 was not stable in *S. thermophilus* after repeated culturing without selective pressure (Fig. 3a). Taken altogether, the presence of AcrIIA5 and AcrIIA6 proteins increased plasmid stability in *S. thermophilus*. Of significant interest, the plasmid stability obtained with AcrIIA6_123_ and AcrIIA6_D1811-Δ59_ was a remarkable result since we had shown that the interference activity of Cas9 was not blocked by the truncated ACR. These results suggested that truncated ACRs may prevent spacer acquisition.

Since plasmid loss assays were conducted by serial passages of bacterial cultures in liquid medium without selective antibiotic pressure (to favor plasmid loss), we performed PCR amplifications of the CR1 array directly on these *S. thermophilus* cultures (Fig. 3b) and performed Illumina sequencing on the resulting PCR products to estimate the spacer acquisition rate. We focused our analysis on the CR1 locus because most of the spacer acquisition events occur in this array in strain DGCC7710^20^, and more importantly, the interference activity of its associated Cas9 is blocked by AcrIIA5 and AcrIIA6. Through the analysis of the sequencing reads, we detected spacer acquisition in 33% of the reads from the culture of *S. thermophilus* carrying the control plasmid pNZ123. In contrast, spacer acquisition was detected in <1% of the reads in cultures containing a plasmid carrying AcrIIA6_D1811_, AcrIIA6_DT1_, or AcrIIA6_123_ (Figs. 3a, S10 and S11). These data confirmed that spacer acquisition was reduced in the presence of ACRs, including the truncated AcrIIA6_123_ that does not block Cas9 interference.

Then, we searched for the targets of the newly acquired spacers. Since the vast majority of acquired spacers targeted the plasmid (Fig. 3c), we mapped them on the plasmid sequence (Fig. 3d). We did not observe a clear preference for any specific acquired spacers. When AcrIIA6_DT1_, AcrIIA6_123_, AcrIIA6_D1811_, or AcrIIA6_D1811-Δ59_ was present, very few spacers were acquired (although more spacer acquisition was observed for AcrIIA6_D1811-Δ59_), and the target locations were spread out along the plasmid sequence. In addition, several spacers targeting the plasmid-based *acr* gene were observed in the cultures containing AcrIIA5_D1126_, AcrIIA6_DT1_, or AcrIIA6_123_. Next, we examined the protospacer-adjacent motifs (PAMs) found on pNZ123 and derivatives to investigate whether they matched the previously predicted PAM (NNAGAAW^21^) associated to the CR1 in this *S. thermophilus* strain. Analysis of the extracted motifs (7 nucleotides downstream from the protospacer, Fig. 3e) in our selection-free experiment revealed the same motif, NNAGAAW. Thus, the presence of a full or truncated Acr did not affect the PAM specificity.

**Fig. 4:**
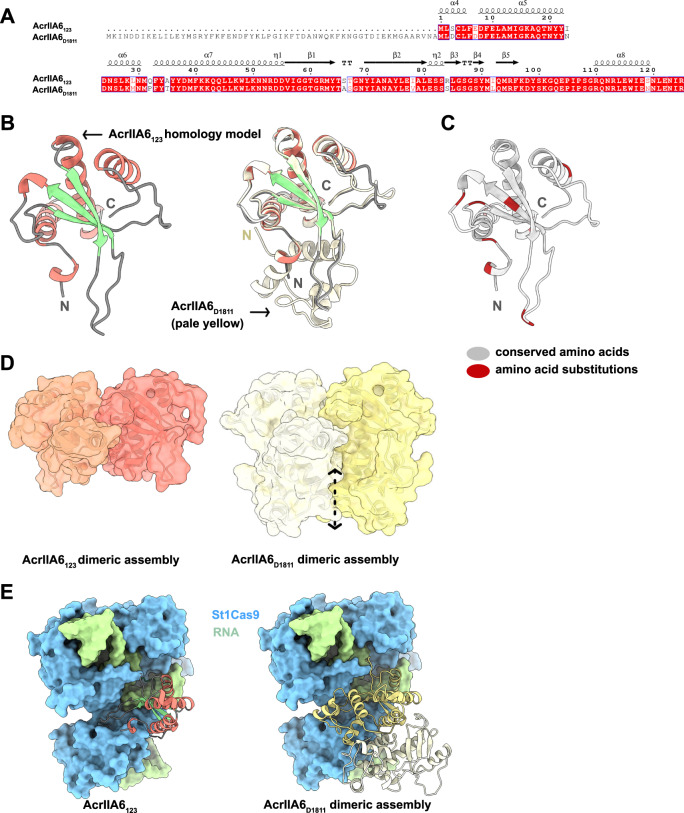
Homology model of AcrIIA6_123_. **A** Sequence alignment of AcrIIA6_123_ and AcrIIA6_D1811_ (residues 59–183). Secondary structure elements from the crystal structure of AcrIIA6_D1811_ are shown above the alignment. **B** Ribbon representation of the AcrIIA6_123_ homology model (left, colors representing secondary structure elements) superimposed on the crystal structure of AcrIIA6_D1811_ (right, pale yellow). **C** Representation of sequence variations between AcrIIA6_123_ and AcrIIA6_D1811_. Gray areas represent 100% sequence identity. Red patches represent amino acid substitutions. **D** Surface representations of AcrIIA6_123_ (left) and AcrIIA6_D1811_ (right) dimeric assemblies. The dotted arrow indicates the dimerization interface missing in the AcrIIA6_123_ dimer. **E** Comparison between the simulated binding interface of AcrIIA6_123_ to St1Cas9-RNA complex (left) and the experimentally demonstrated binding of AcrIIA6_D1811_ to St1Cas9-RNA complex (right).

The above data clearly indicate that AcrIIA6_123_ blocks the CRISPR-Cas adaptation step but not the interference step. We previously determined the crystal structure of AcrIIA6_D1811_^22^, which shares 90% sequence identity with AcrIIA6_123._ We generated a homology model using the AcrIIA6_D1811_ 3D structure as a template (Fig. 4A, B). This 3D model of AcrIIA6_123_ highlights amino acid substitutions distributed over the entire structure (Fig. 4C). As AcrIIA6_D1811_ forms dimers in solution, we produced an AcrIIA6_123_ dimeric model, using the AcrIIA6_D1811_ dimer as a template, and analyzed it using the PISA server^23^. The dimerization interface of AcrIIA6_123_ was reduced by 56% as compared with that of AcrIIA6_D1811_ (Fig. 4D). In addition, the assembly is predicted to be thermodynamically unstable, which indicates that this short version of the AcrIIA6 protein very likely does not form dimers in solution.

We previously showed that the long and dimeric version of AcrIIA6 is an allosteric inhibitor of St1Cas9 and that it strongly binds to the protein-RNA surface of the St1Cas9-crRNA complex, thereby preventing interference^24^. Interestingly, the monomeric AcrIIA6_123_ presents two notable features by (1) offering a much smaller binding surface than that of the dimeric AcrIIA6, and (2) containing an amino acid substitution (I23 in AcrIIA6_123_ instead of N81 in AcrIIA6) that likely disrupts the St1Cas9-RNA-binding interface. Based on these features, AcrIIA6_123_ likely does not bind to the St1Cas9-RNA surface in a stable manner. Whether AcrIIA6_123_ could bind to another region of the St1Cas9-RNA complex, to St1Cas9 only, or even to another component of the spacer acquisition machinery, the mechanism of this anti-acquisition activity remains to be determined. Even though the Cas1-Cas2 complex has been shown to select spacer precursors^22^ and Cas9 is involved in spacer acquisition^25^, gaps remains to understand the molecular mechanisms of spacer integration in itself^26–28^.

In conclusion, we were able to maintain the interference activity of the CRISPR-Cas system against ACR+ phages with a spacer directly targeting their *acr* genes. Phage amplification on these resistant strains led to the isolation of phage-escaping mutants with truncated *acr* genes that are no longer targeted by the spacer but also cannot block the interference activity of Cas9. The outcome of this arms race between phage and bacteria may explain the possible origins of the short ACRs. Interestingly, the presence of a short AcrIIA6 was still able to prevent spacer acquisition. As spacer integration is still the less understood step in the biology of CRISPR-Cas systems, it is believed that the identification of inhibitors of this process will provide novel tools to study this molecular mechanism. Finally, we also show that the presence of *acr* genes significantly increases plasmid stability within bacterial strains possessing active CRISPR-Cas systems. Increased plasmid stability through an ACR may provide a significant advantage in biotechnological applications. It also paves the way for the discovery of other anti-acquisition ACRs that may play a role in the persistence and maintenance of mobile genetic elements carrying various phenotypes such as antibiotic resistance or virulence genes^29–32^.

## Methods

### Phage amplification and titration

Scrapings of phage lysates stored at −80 °C with 15% glycerol were co-inoculated and incubated at 42 °C with their host strain in LM17 medium supplemented with 10 mM of CaCl_2_ (LM17-Ca) until complete lysis. The lysates were then filtered (0.45 μm PES filter) and 100 μl of the filtrate was used to inoculate the host strain, grown to an OD_600_ of 0.1. The second amplification lysate was also similarly filtered and stored at 4 °C. Phage titers were obtained through serial dilutions of phage lysates in buffer (50 mM Tris-HCl, pH 7.5, 100 mM NaCl, 8 mM MgSO_4_). One-hundred microliters of diluted phage lysate and 300 μl of an indicator strain grown to an OD_600_ of 0.6 were co-inoculated into 3 ml of molten 0.75% agar LM17-Ca at 55 °C. The molten mix was immediately poured onto a plate of LM17-Ca with 1% agar and allowed to set. The plates were incubated overnight at 42 °C, and plaques were counted on those plates. Dilutions resulted in plates containing between 30 and 300 plaques were used to estimate the phage titers.

### Phage genome sequencing and annotation

Sequencing libraries were prepared with the Nextera XT DNA Library Preparation Kit (Illumina) according to the manufacturer’s instructions. The library was sequenced using a MiSeq Reagent Kit V2 (Illumina, 500 cycles) on a MiSeq system, using paired-ends (2 × 250 bp). De novo assembly was performed with Ray assembler version 3.0.0^33^. Nucleotide coverage was calculated with SAMtools^34^. The sequences were analyzed using Geneious software version 11.1.5. Open reading frames were identified with GeneMark.hmm^35^. A sequence was considered an ORF only if its starting codon was AUG, UUG, or GUG and it possessed at least 30 amino acids. The BLASTp database was used to predict the function of each ORF.

### BIM generation

BIMs of *S. thermophilus* UY04 were obtained following an infection with phage 123 as described previously^36^. Briefly, the host UY04 was grown to an OD_600_ of 0.6 and was then added to a molten 0.75% agar with a diluted lysate of phage 123. Incubation was done for ~18 h at 37 °C and an additional 24 h at 42 °C before the growth of phage-resistant colonies. Spacer acquisition in CR1 and CR3 were verified using two sets of primers, Yc70 and CR1-UY04-intern-R (CR1) and CR3-fwd and CR3-UY04-intern-R (CR3) (Supplementary Table 1).

### Anti-CRISPR protein alignments

Truncated ACRs were searched in NCBI database (January 2022) by tBLASTn using known representatives of AcrIIA. We restricted our criteria to truncated Acr homologs to share at least 50% of amino-acid identity on the BLAST aligned section, and a BLAST e-value <0.001. Sequences from uncultured isolates were excluded. Acr truncated homologs were then manually verified to confirm that they were predicted ORFs, including start and stop codons as well as at least 30 amino acids long but not exceeding 90% of length of the representative Acr allele. Examination of the genomic context for the predicted ORFs, in plasmids and/or (pro)phages, was performed manually. Protein alignments were performed with Geneious software version 11.1.5.

### Plasmid constructs

Primers were designed to clone the *acr* gene of phage 123 (Supplementary Table 1), containing 30-nt extensions that overlap with the pNZ123 multiple cloning site to facilitate Gibson assembly^10,11^. The amplified gene was then cloned by Gibson reaction into pNZ123 that was linearized after digestion with XbaI. The resulting plasmid was transformed into commercial NEB5α *E. coli* cells, according to the manufacturer’s recommendations (New England Biolabs). The resulting plasmid was purified using a Qiaprep Spin Miniprep kit (Qiagen). The purified plasmids were then electroporated into the relevant *S. thermophilus* strains, that were made competent through a glycine shock-based protocol^36^. The inserts were confirmed by Sanger sequencing using primers pNZins_F and pNZins_R (Supplementary Table 1).

A truncated version of AcrIIA6_D1811_ was obtained from the plasmid pNZAcrD1811 in order to create a deletion of the first 59 amino acids. To obtain amplicons, PCR reactions were performed with the appropriate primers (Supplementary Table 1) on a colony containing pNZAcrD1811 with the Q5 polymerase (New England Biolabs). PCR products were purified on a 0.8% agarose gel using the QIAquick PCR Purification kit (Qiagen), following the manufacturer’s instructions. Then, phosphorylation at the 5′ end of the amplicon was performed using 500 ng of purified PCR product, in the T4 DNA Ligase Reaction Buffer 5X (New England Biolabs) and 10 U/µl of Polynucleotide kinase (Roche). A ligation reaction was performed with 100 ng of the phosphorylation product, in the T4 DNA Ligase Reaction Buffer 5X and the T4 DNA Ligase. The ligation products were transformed in chemically competent *E. coli* NEB5α cells and transformants were selected on LB plates with 20 µg/ml chloramphenicol. Constructions were confirmed by PCR and Sanger sequencing. Plasmids were then extracted using a Qiaprep Spin Miniprep kit and electroporated into *S. thermophilus*^36^. Transformants were selected on LM17 plates with Cm 5 and confirmed by PCR and Sanger sequencing.

### Plasmid assays

Unless otherwise stated, *S. thermophilus* strains carrying different plasmids (pNZ123/pNZAcr) were grown overnight at 37 °C in M17 broth (Oxoid) with 0.5% w/v lactose (LM17) and 5 μg/ml chloramphenicol (Cm 5). Then, 100 μl of the overnight culture was used to inoculate 10 ml of the LM17 broth culture (selection-free). Once the cultures were grown to stationary growth phase, they were serially transferred (1%) into fresh media, with a total of 9 transfers in the absence of antibiotic selection. Cultures were then diluted, plated on LM17, and incubated overnight at 42 °C to obtain isolated colonies. For each treatment, 100 single colonies were randomly selected and screened on plates of LM17, either with or without antibiotics, and incubated overnight at 42 °C. Any colony that grew in LM17 but not in LM17 Cm 5 plates was considered to have lost the plasmid. Those colonies were then screened for expansion of the CRISPR arrays by PCR. Primers are listed in Supplementary Table 1.

### CRISPR arrays expansion and deep-sequencing of CR1 loci

The CRISPR loci of *S. thermophilus* cultures were directly amplified by PCR. Then, libraries for high-throughput sequencing of PCR products were prepared following the “16S Metagenomic Sequencing Library Preparation” protocol by Illumina, using primers with extensions that contain the adapters and the MiSeq Reagent Kit v2 (Supplementary Table 1). Libraries were sequenced on an Illumina MiSeq apparatus. Paired reads were assembled using PandaSeq^37^ and further data analysis was conducted on fasta files in Python. First, expanded CRISPR arrays were identified researching repeat sequences, which detected the number of spacer-repeat sequences. Subsequent newly acquired spacer sequences were then extracted and their target location was assessed by performing BLASTn on reference sequences.

### AcrIIA6 homology model

We generated the AcrIIA6_123_ homology model using MODELLER in the frame of the MPI Bioinformatics tool kit^38,39^. We analyzed this model using the PISA server^23^ and prepared figures using ESPript^40^ and ChimeraX^41^. ChimeraX was developed by the Resource for Biocomputing, Visualization, and Informatics at UCSF with support from NIH R01-GM129325 and the Office of Cyber Infrastructure and Computational Biology, NIAID.

### Reporting summary

Further information on research design is available in the Nature Research Reporting Summary linked to this article.

## Supplementary information

Supplementary Information

Reporting Summary

## Data Availability

Accession numbers from NCBI for phage genomes and genes studied in this article are provided in the text and are publicly available. High-throughput sequencing raw reads generated from Illumina are available through NCBI SRA repository under the study number SRP369920 and bioproject accession number PRJNA826091. Python code used to extract and count spacers is available on github (https://github.com/edzuf/Truncated_ACR). Source data are provided as a Source data file. The authors declare that all other data supporting the findings of this study are available within the article and its Supplementary Information files, or are available from the authors upon request. Source data are provided with this paper.

## Source data

Source Data
