# Author Correction: A truncated anti-CRISPR protein prevents spacer acquisition but not interference

**DOI:** 10.1038/s41467-022-31631-7

**Published:** 2022-07-05

**Authors:** Cécile Philippe, Carlee Morency, Pier-Luc Plante, Edwige Zufferey, Rodrigo Achigar, Denise M. Tremblay, Geneviève M. Rousseau, Adeline Goulet, Sylvain Moineau

**Affiliations:** 1 Département de biochimie, microbiologie, et bio-informatique, Faculté des sciences et de génie, Université Laval, Québec City, QC Canada; 2 Groupe de recherche en écologie buccale, Faculté de médecine dentaire, Université Laval, Québec City, QC Canada; 3 Département de médecine moléculaire, Faculté de médecine, Université Laval, Québec City, QC Canada; 4 Laboratorio de Microbiología Molecular, Departamento de Biociencias, Facultad de Química, Universidad de la República, Montevideo, Uruguay; 5 Félix d’Hérelle Reference Center for Bacterial Viruses, Faculté de médecine dentaire, Université Laval, Québec City, QC Canada; 6 Laboratoire d’Ingénierie des Systèmes Macromoléculaires, Institut de Microbiologie, Bioénergies et Biotechnologie, CNRS UMR7255, Aix-Marseille Université, Marseille, 13402 France

**Keywords:** Phage biology, Bacteriophages, Bacteria, Virus-host interactions

Correction to: *Nature Communications* 10.1038/s41467-022-30310-x, published online 19 May 2022.

The original version of this Article contained the following errors:

It contained an error in Fig. 4A, in which a 6-amino acid insertion (positions 101–106) was incorrectly shown in protein AcrIIA6 123, compared to AcrIIA6 D1811. The correct figure now shows that this 6-amino acid sequence is also present in AcrIIA6 D1811.

It contained an error in Fig. 4C, in which a 6-amino acid insertion (positions 101–106) was incorrectly highlighted using green colour. This highlighting has been removed from the figure.

It contained an error in Fig. 4E, in which a 6-amino acid insertion (positions 101–106) was incorrectly highlighted using a dotted ellipse. The dotted ellipse has been removed from the figure.

It contained errors in the legend of Fig. 4, which incorrectly read ‘Red and green represent amino acid substitutions and amino acid insertions, respectively’ and ‘The dotted ellipse highlights the position of the amino acid insertion in AcrIIA6_123_’. The correct version replaces the first sentence with ‘Red patches represent amino acid substitutions’, and removes the second sentence.

It contained an error in a sentence of the ‘Results and discussion’ section, which incorrectly read ‘This 3D model of AcrIIA6_123_ highlights a 6-residue insertion (K101-I106) in the loop connecting the β-sheet to the C-terminal α-helix, as well as some amino acid substitutions distributed over the entire structure’. The correct version replaces this sentence with ‘This 3D model of AcrIIA6_123_ highlights amino acid substitutions distributed over the entire structure’.

It contained an error in a sentence of the ‘Results and discussion’ section, which incorrectly read ‘Interestingly, the monomeric AcrIIA6_123_ presents three notable features by (1) offering a much smaller binding surface than that of the dimeric AcrIIA6, (2) harboring the amino acid insertion in an RNA-interacting loop, and (3) containing an amino acid substitution (I23 in AcrIIA6_123_ instead of N81 in AcrIIA6) that likely disrupts the St1Cas9-RNA-binding interface’. The correct version replaces this sentence with ‘Interestingly, the monomeric AcrIIA6_123_ presents two notable features by (1) offering a much smaller binding surface than that of the dimeric AcrIIA6, and (2) containing an amino acid substitution (I23 in AcrIIA6_123_ instead of N81 in AcrIIA6) that likely disrupts the St1Cas9-RNA-binding interface’.

The errors have been corrected in both the PDF and HTML versions of the Article.

