# Supplementary material for: A truncated anti-CRISPR protein prevents spacer acquisition but not interference
Source: Nat Commun. 2022 May 19;13:2802. doi: 10.1038/s41467-022-30310-x (PMC9120153; doi:10.1038/s41467-022-30310-x)
Supplement: Supplementary file 1 — Supplementary Information [file 41467_2022_30310_MOESM1_ESM.pdf]

Supplementary Figures

a

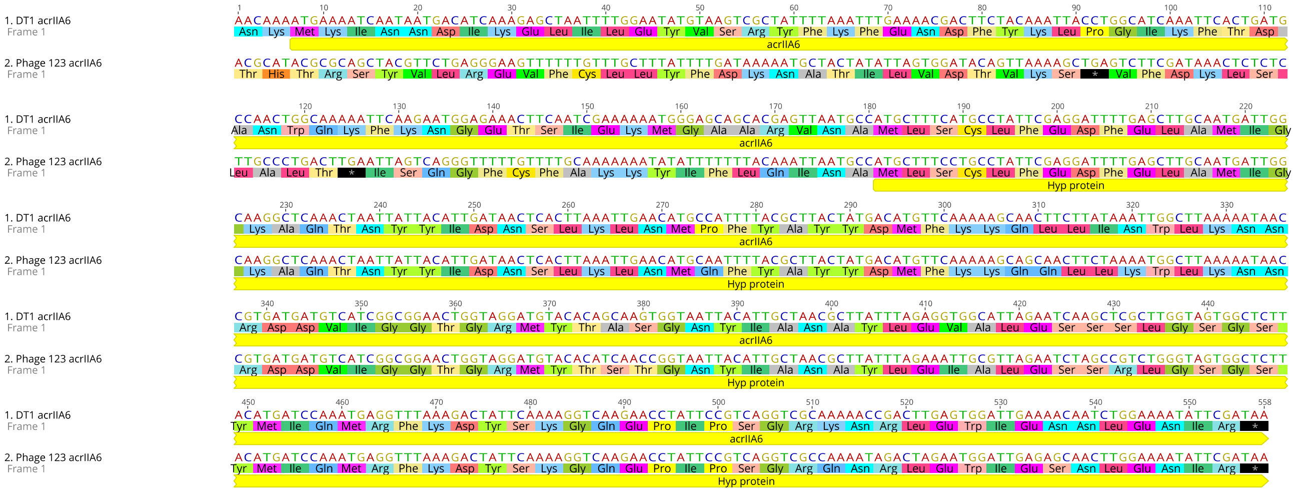

b

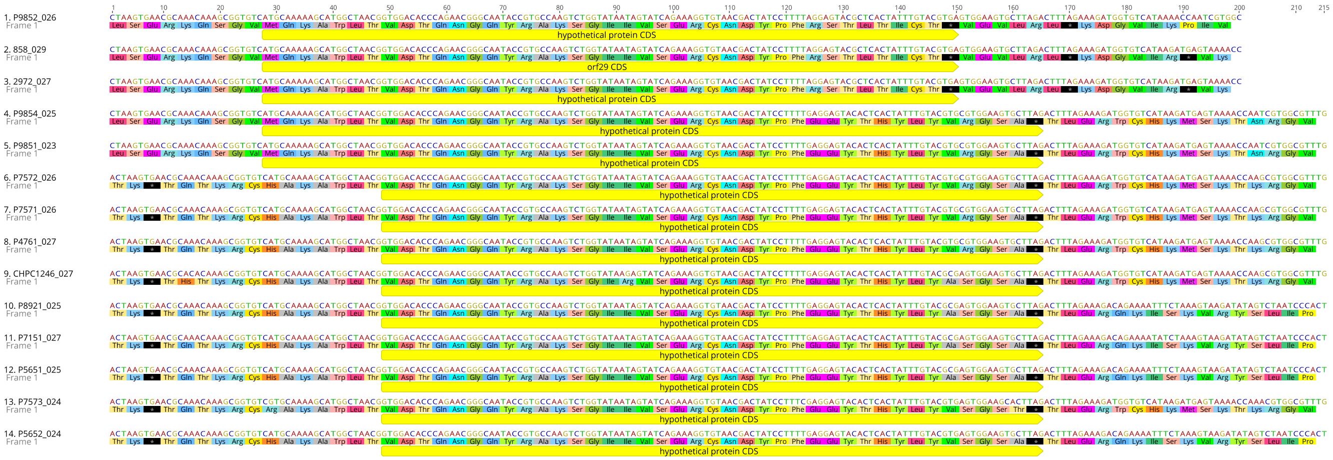

c

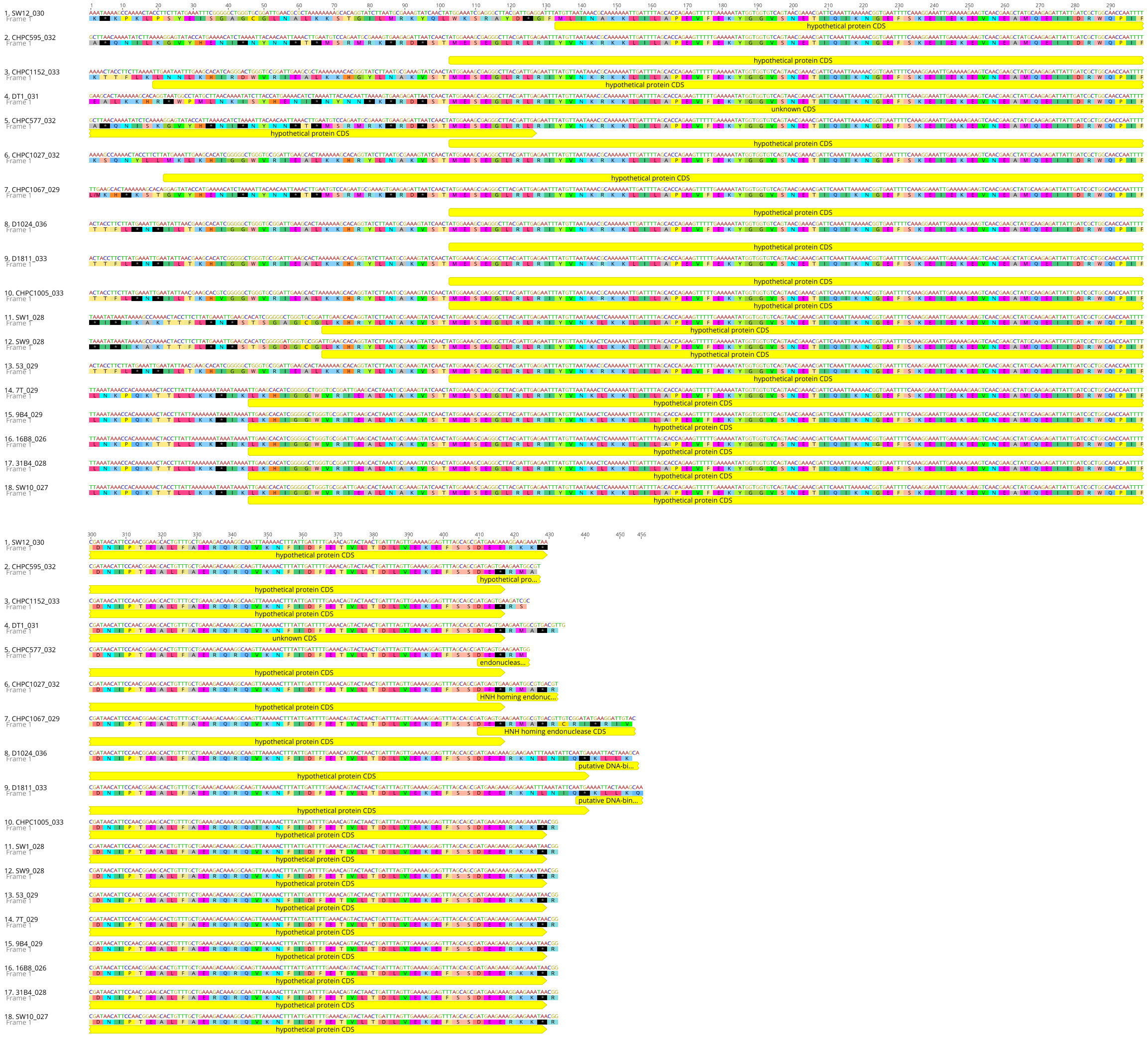

Figure S1. Sequence alignment of non-essential phage ORFs from the replication module. (a) AcrIIA6 from phage 123 in this study and related allele AcrIIA6 from phage DT1. (b) ORF27$_{2972}$ homologs. (c) ORF31$_{DT1}$ homologs. Alignments of nucleotides and amino-acid sequences illustrate the presence of mutations and deletions leading to change in the start codon and protein truncation in this genomic region.

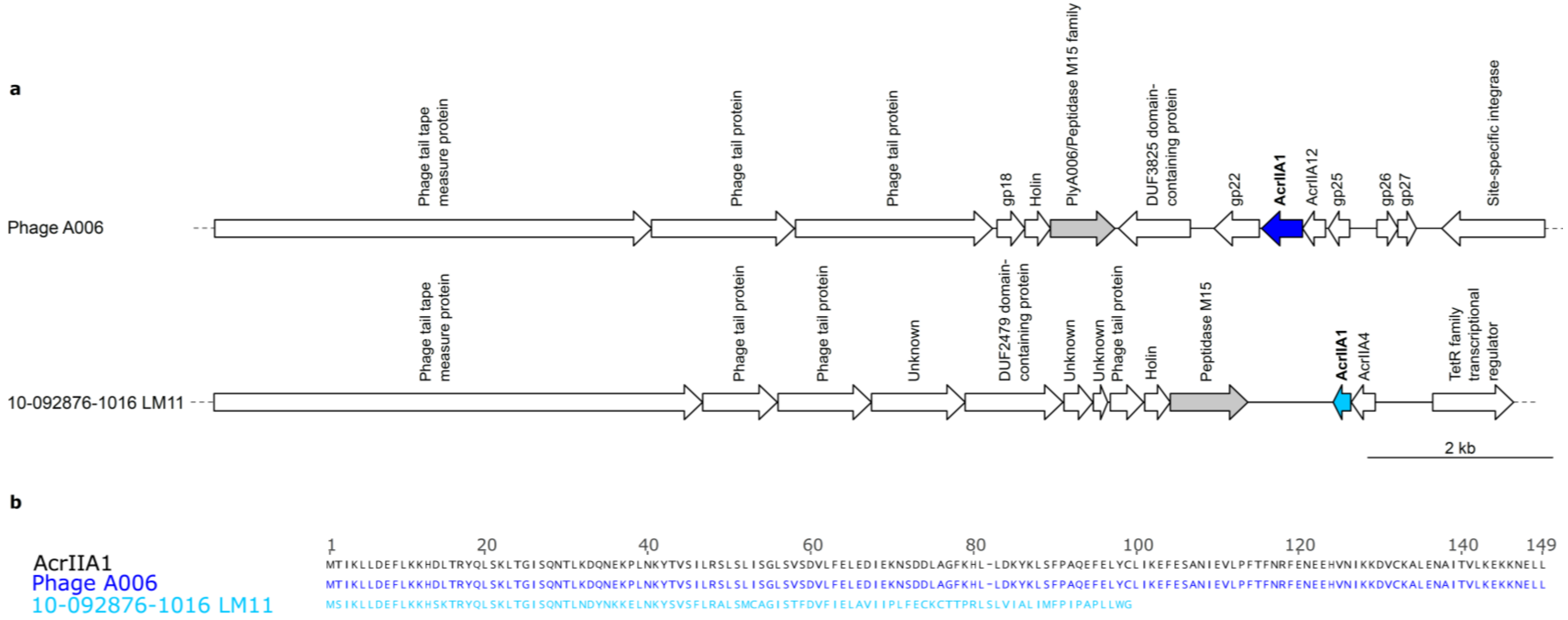

Figure S2. Genome context and sequence alignment of AcrIIA1 and truncated versions in *Listeria monocytogenes* strain or phage. **a**. Comparison of genomic regions of one *L. monocytogenes* strain and one *L. monocytogenes* temperate phage. The genomic positions 9,308 to 23,910 are represented in the first row for the genome of *L. monocytogenes* phage A006 and positions 2,663,071 to 2,648,805 are shown in the second row for *L. monocytogenes* strain 10-092876-1016 LM11. Arrows represent ORFs and light grey arrows indicate at least 40% identity in amino acids between homologous proteins. The dark blue arrow highlights a full-length AcrIIA1 present in the genome of *L. monocytogenes* phage A006. The light blue arrow shows a truncated AcrIIA1 in *L. monocytogenes* strain 10-092876-1016 LM11. **b**. Amino acids alignment of AcrIIA1 and one truncated version found with tblastn analyses. Strain or phage name/GenBank accession/locus tag or product: A006/DQ003642/gp23; 10-092876-1016 LM11/CP019624/B0X17_13420.

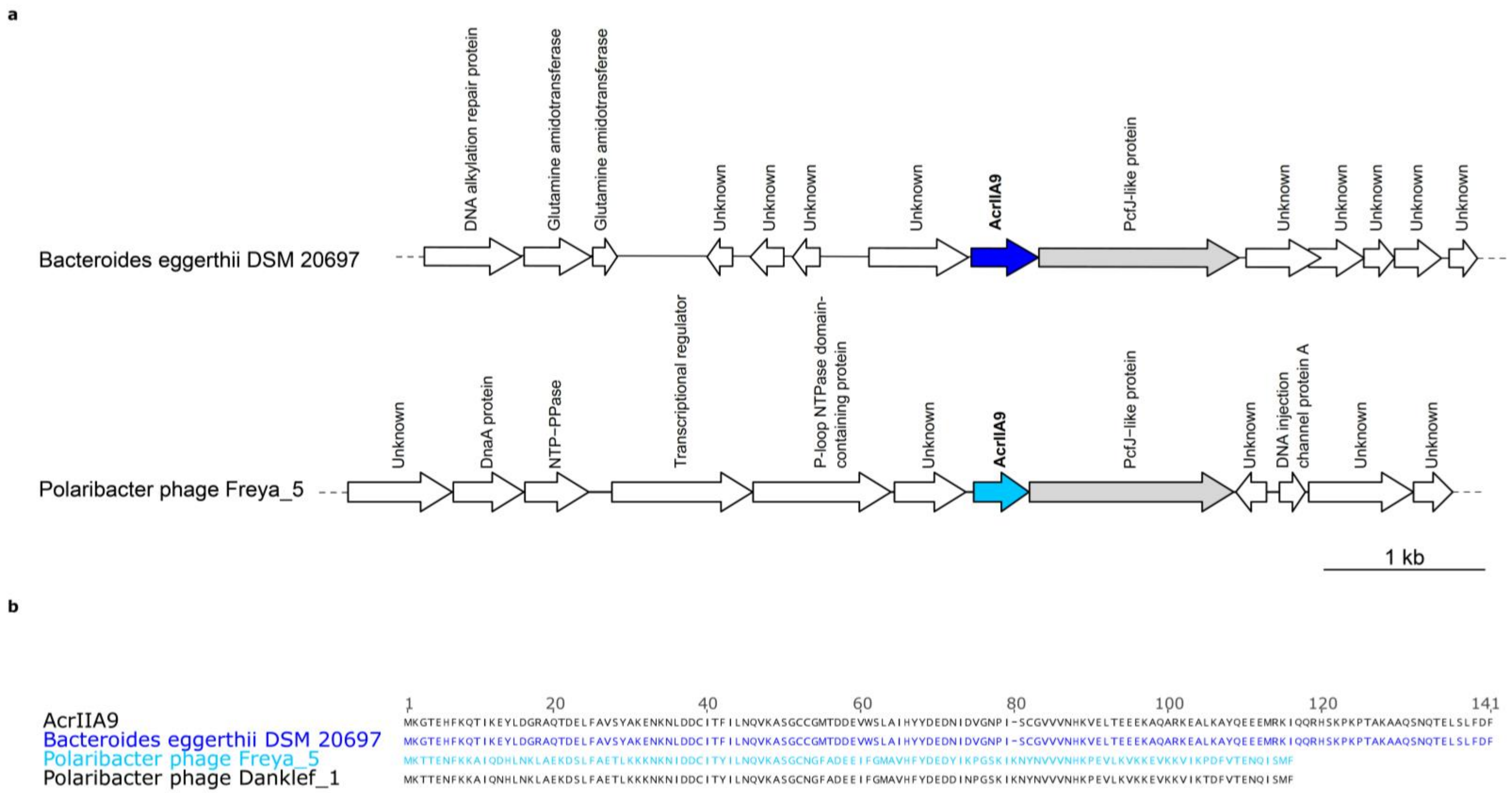

Figure S3. Genome context and sequence alignment of AcrIIA9 and truncated versions in *Bacteroides eggerthii* strain or in *Polaribacter* sp. phages. **a**. Comparison of genomic regions of a *B. eggerthii* strain and a *Polaribacter* phage. The genomic positions 89,635 to 82,975 are represented in the first row for the contig of *B. eggerthii* DSM 20697 and positions 35,540 to 42,527 are shown in the second row for *Polaribacter* phage Freya_5. Arrows represent ORFs and light grey arrows indicate at least 30% identity in amino acids between homologous proteins. The dark blue arrow highlights a full-length AcrIIA9 present in the genome of *B. eggerthii* DSM 20697. The light blue arrow shows a truncated AcrIIA9 in phage Freya_5. **b**. Amino acids alignment of AcrIIA9 and two truncated versions found with tblastn analyses. Strain or phage name/GenBank accession/locus tag: DSM 20697/ABVO01000019/BACEGG_01111; Freya_5/MT732467/Freya5_60; Danklef_1/MT732458/Danklef1_46.

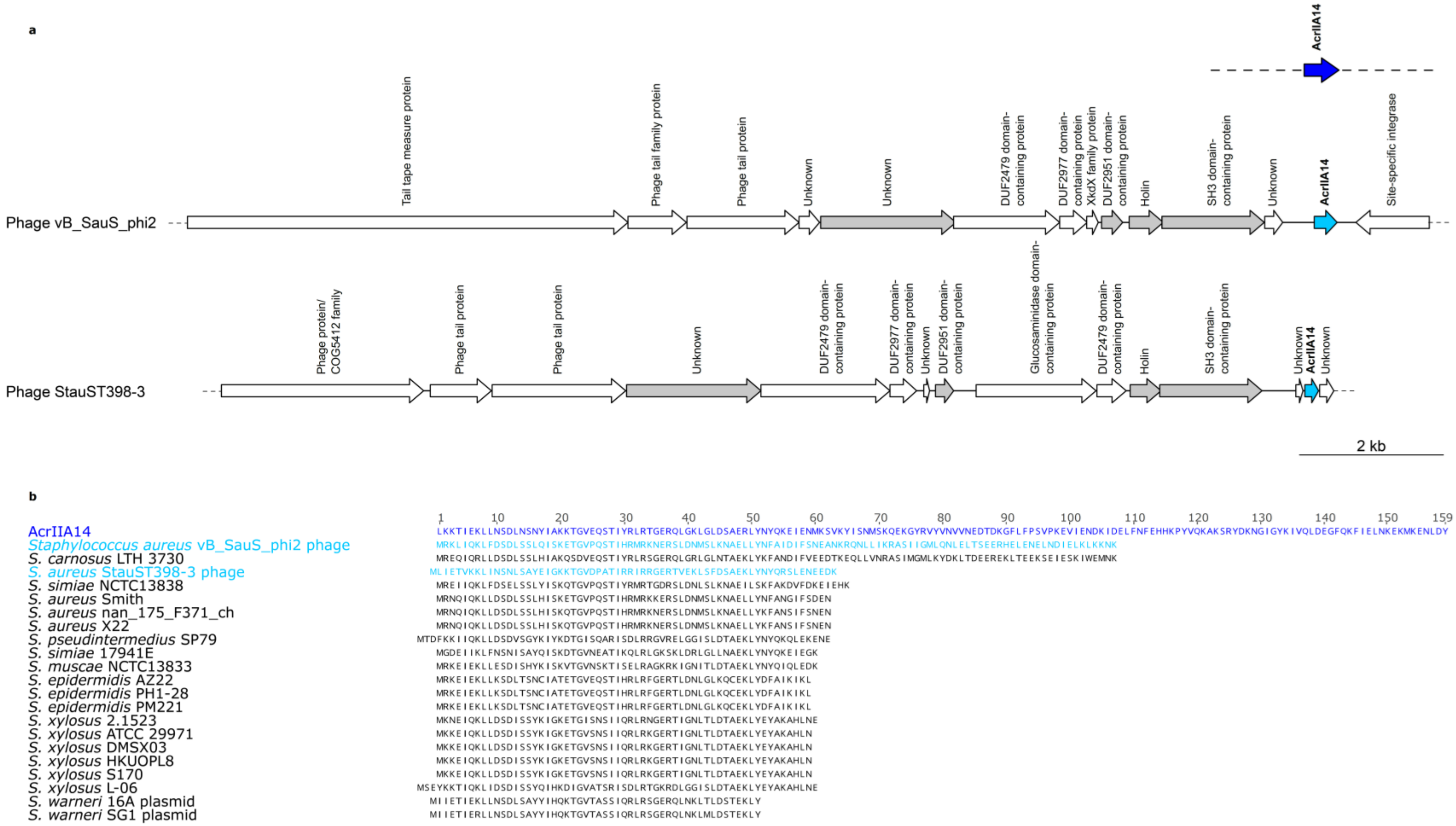

Figure S4. Genome context and sequence alignment of AcrIIA14 and examples of truncated versions in *Staphylococcus* strains, phages or plasmids. **a**. Comparison of genomic regions of two *S. aureus* phages. The genomic positions 8,514 to 26,058 are represented in the first row for *S. aureus* phage vB_SauS_phi2 and positions 25,499 to 41,214 are shown in the second row for *S. aureus* phage StauST398-3. Arrows represent ORFs and light grey arrows indicate at least 40% identity in amino acids between homologous proteins. The dark blue arrow highlights the full-length model AcrIIA14 as no genomic context was found for the 159 amino acids version. The light blue arrows show truncated AcrIIA14 in two *S. aureus* phages. **b**. Amino acids alignment of AcrIIA14 and some of the many truncated versions found with tblastn analyses. Strain, phage or plasmid name/GenBank accession/locus tag when available: vB_SauS_phi2/NC_028862/AVU17_gp26; LTH 3730/CP016760/BEK99_04395; StauST398-3/JQ973847/StauST398-3_0068;

NCTC13838/LT906460/SAMEA4384339_01206; Smith/CP029751/CFC57_06345; nan_175_F371_ch/CP066492/JF380_05885; X22/CP042650; SP79/AP019372/GSP_15320; 17941E/CP071589/J3R86_07905; NCTC13838/LT906460/SAMEA4384339_01206; AZ22/CP071994; PH1-28/CP066376; PM221/HG813242/SEB_00586; 2.1523/CP066721/JGY88_09280; ATCC 29971/LT963439; DMSX03/CP060271; HKUOPL8/CP007208/BE24_06610; S170/CP013922/AWC37_06820; L-06/LN795825; 16A/CP031267/DWB88_00555; SG1/CP003671/A284_12087.

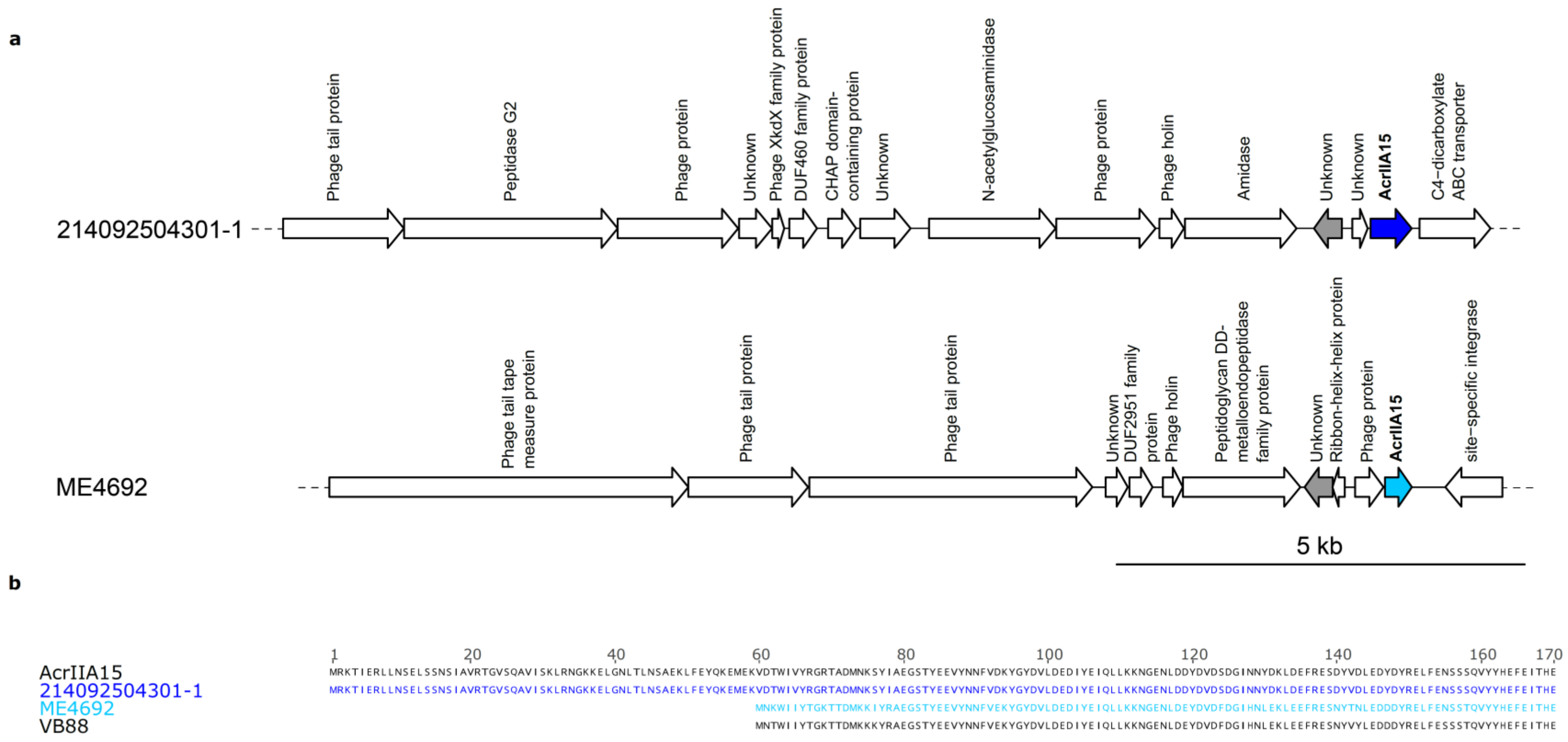

Figure S5. Genome context and sequence alignment of AcrIIA15 and truncated versions in *Staphylococcus* strains. **a**. Comparison of genomic regions of a *S. delphini* and a *S. pseudintermedius* strains. The genomic positions 21 to 28,042 are represented in the first row for a contig of *S. delphini* strain 214092504301-1 and positions 1,176,583 to 1,190,467 are shown in the second row for *S. pseudintermedius* strain ME4692. Arrows represent ORFs and grey arrows indicate at least 80% identity in amino acids between homologous proteins. The dark blue arrow highlights a full-length AcrIIA15 present in the genome of *S. delphini* strain 214092504301-1. The light blue arrow shows a truncated AcrIIA15 in *S. pseudintermedius* ME4692. **b**. Amino acids alignment of AcrIIA15 and 2 truncated versions found with tblastn analyses. Strain name/GenBank accession/locus tag: 214092504301-1/MWUW01000002/B5C05_01750; ME4692/CP039747/EFT29_005745; VB88/CP30715/DNH96_11305.

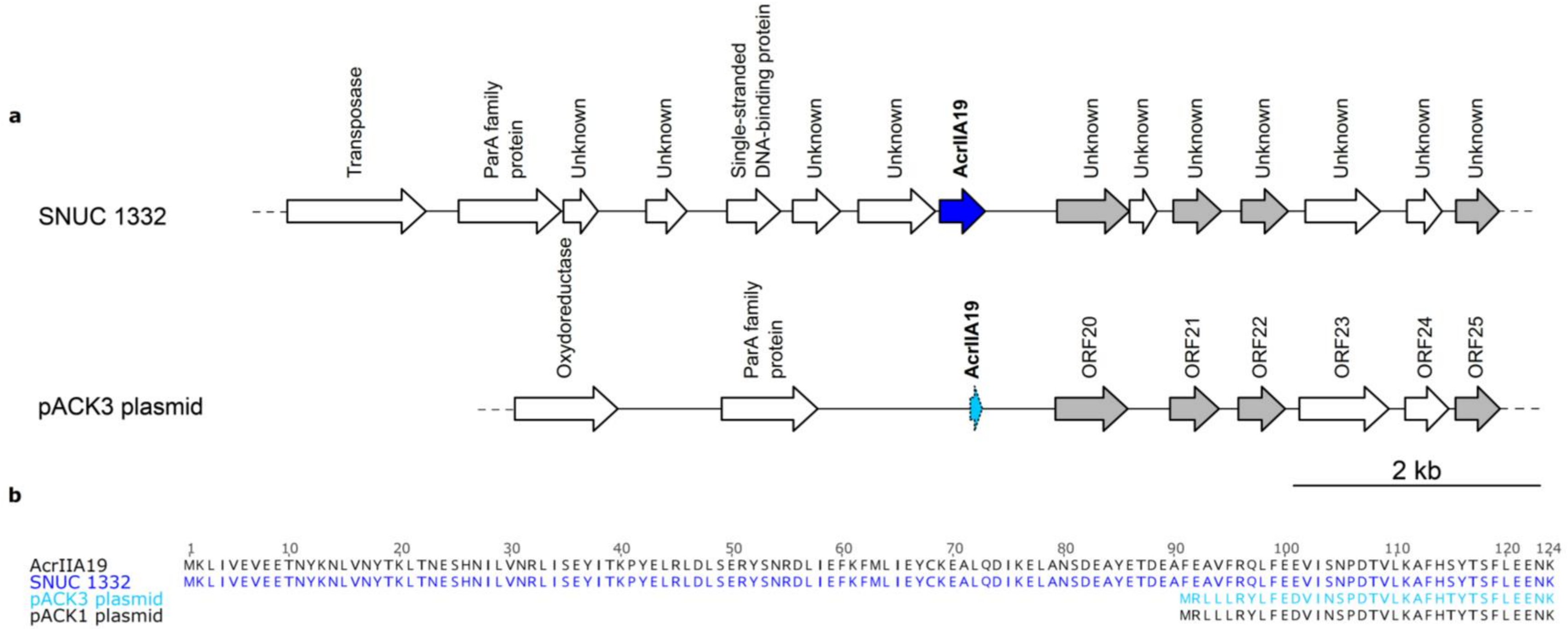

Figure S6. Genome context and sequence alignment of AcrIIA19 and truncated versions in *Staphylococcus simulans* strain or plasmids. **a**. Comparison of genomic regions of one *S. simulans* strain and one *S. simulans* biovar *staphylolyticus* plasmid. The genomic positions 45,416 to 55,400 are represented in the first row for a contig of *S. simulans* strain SNUC 1332 and positions 18,073 to 26,189 are shown in the second row for *S. simulans* biovar *staphylolyticus* plasmid pACK3 of strain NRRL B-2628. Arrows represent ORFs and black arrows indicate at least 50% identity in amino acids between homologous proteins. The dark blue arrow highlights a full-length AcrIIA19 present in the genome of *S. agalactiae* SNUC 1332. The light blue dotted arrow shows a manually annotated truncated AcrIIA19 in *S. simulans* plasmid pCKA3. **b**. Amino acids alignment of AcrIIA19 and 2 truncated versions found with tblastn analyses. All the truncated Acrs represented were not annotated in the database. Strain-plasmid name/GenBank accession/locus tag when available: SNUC 1332/PZGO01000018 contig/BU024_10435; NRRL B-2629 plasmid pACK3/GU228572; NRRL B-2629 plasmid pACK1/GU228571.

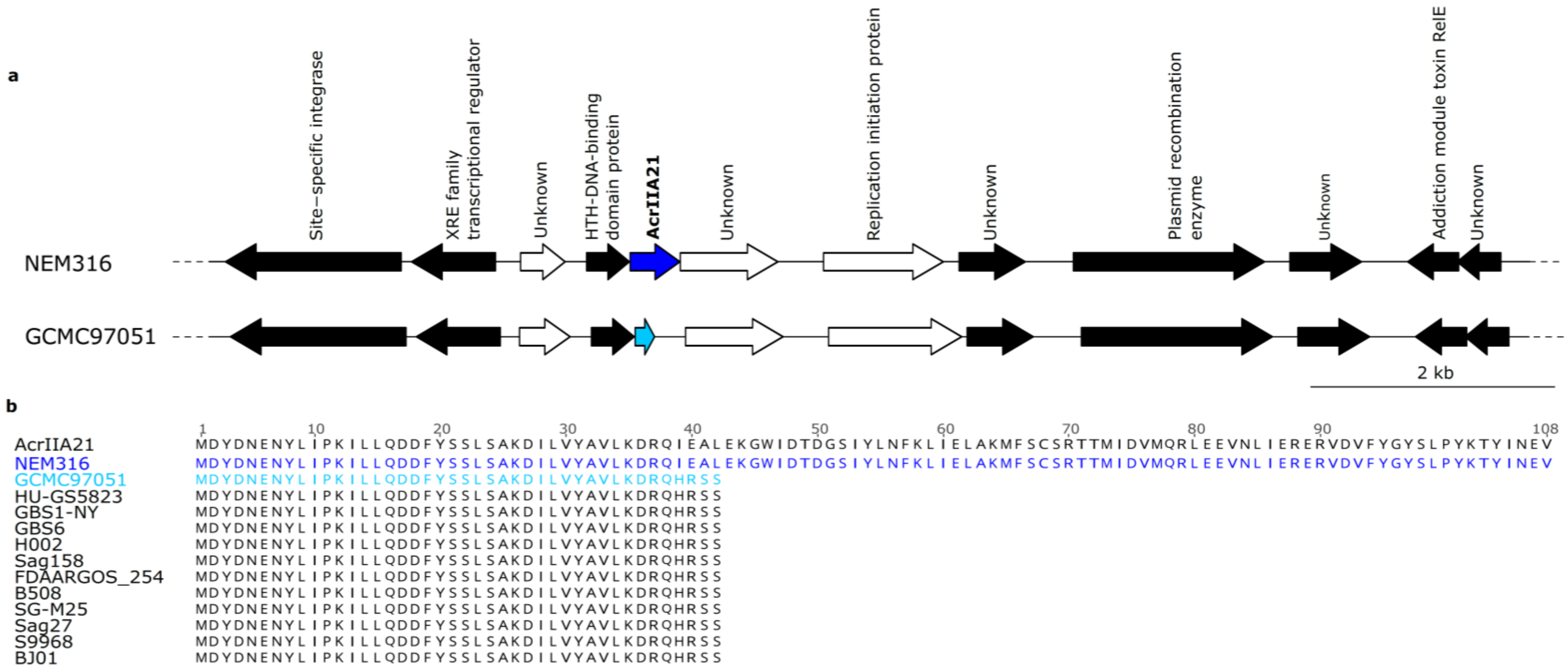

Figure S7. Genome context and sequence alignment of AcrIIA21 and truncated versions in *Streptococcus agalactiae* strains. **a**. Comparison of genomic regions of two *S. agalactiae* strains. The genomic positions 88,729 to 98,536 are represented in the first row for *S. agalactiae* strain NEM316 and positions 288,588 to 298,415 are shown in the second row for *S. agalactiae* strain GCMC97051. Arrows represent ORFs and black arrows indicate 100% identity in amino acids between homologous proteins. The dark blue arrow highlights a full-length AcrIIA21 present in the genome of *S. agalactiae* NEM316. The light blue arrow shows a truncated AcrIIA21 in *S. agalactiae* GCMC97051. **b**. Amino acids alignment of AcrIIA21 and 12 truncated versions found with tblastn analyses. All the truncated Acrs represented here are due to a frameshift mutation. Strain name/GenBank accession/locus tag or gene when available: NEM316/AL766844/gbs0215; GCMC97051/AP020310/SAGCMC97051_03030; HU-GS5823/AP018935/SAGS_0261; GBS1-NY/CP007570; GBS6/CP007572; H002/CP011329; Sag158/CP019979/B2G84_01395; FDAARGOS_254/CP020449/A6J82_00585; B508/CP021770/CDH83_00020; SG-M25/CP021867/ BB164_01590; Sag27/CP031556/DY328_05665; S9968/CP058666/S9968_100411; BJ01/CP059383/H1A19_01360.

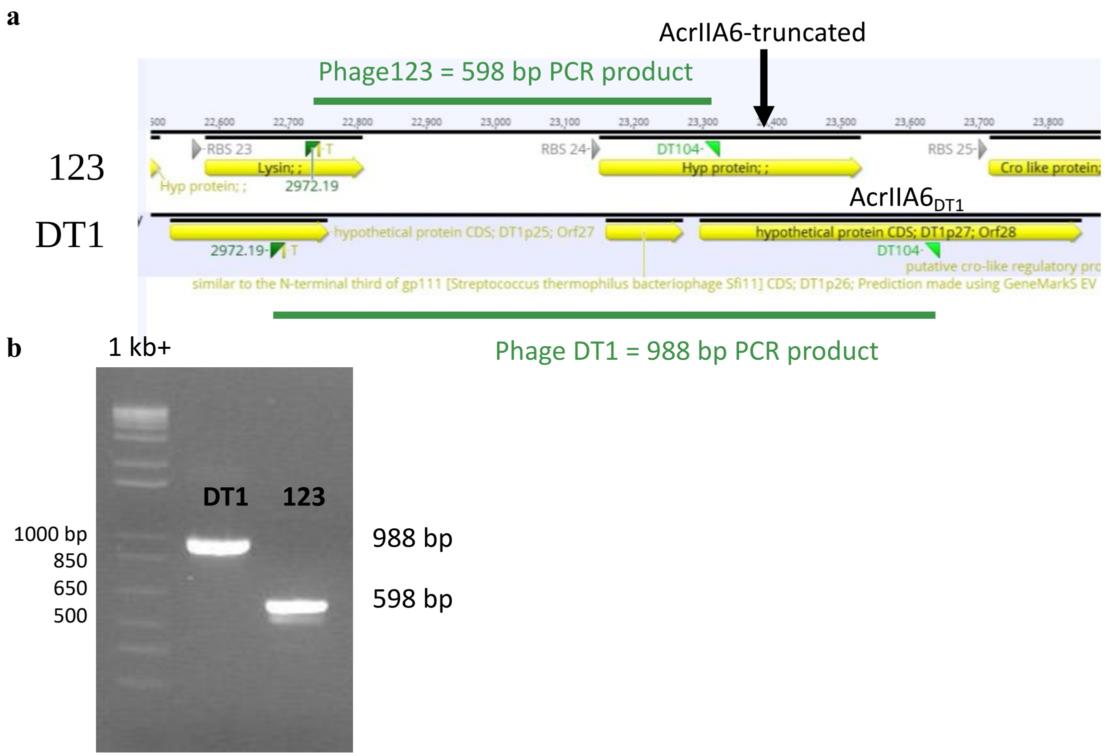

Figure S8. Truncated AcrIIA6 of phage 123 verified by PCR and sequencing. a) Annotation of *acrIIA6* regions in phages 123 and DT1, with positions of primers used for the PCR and predicted RBS of the truncated AcrIIA6. b) Different sizes of amplicons confirmed genome sequencing of phage 123 and the presence of the truncated AcrIIA6 (representative of 2 independent replicates)

a

Phage/Host	Spacer acquisition in CR1	Spacer acquisition in CR3
123/UY04	0/20	20/20

b

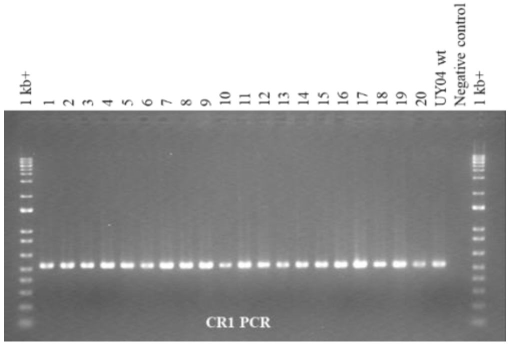

c

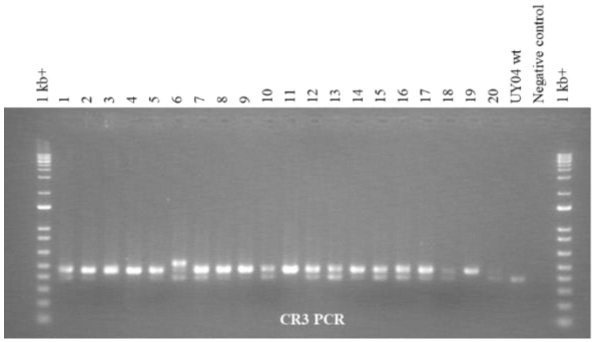

Figure S9. ***S. thermophilus*** **wild-type strain UY04 do not acquire new spacers in its CR1 array when infected with phage 123, which contain a truncated AcrIIA6.** a) Spacer acquisition was evaluated by PCR on colonies of Bacteriophage-Insensitive Mutants (BIMs) obtained after the infection of the wild-type *S. thermophilus* strain UY04 with the virulent phage 123. Summary of the number of BIMs tested and the number of BIMs that have acquired new spacers in the CR1 and the CR3 loci of this strain. b) PCR amplification of the 5' end of the CR1 locus on 20 BIMs and the wild-type strain. c) PCR amplification of the 5' end of the CR3 locus on the same 20 BIMs and the wild-type strain. The gels are representative of two independant replicates.

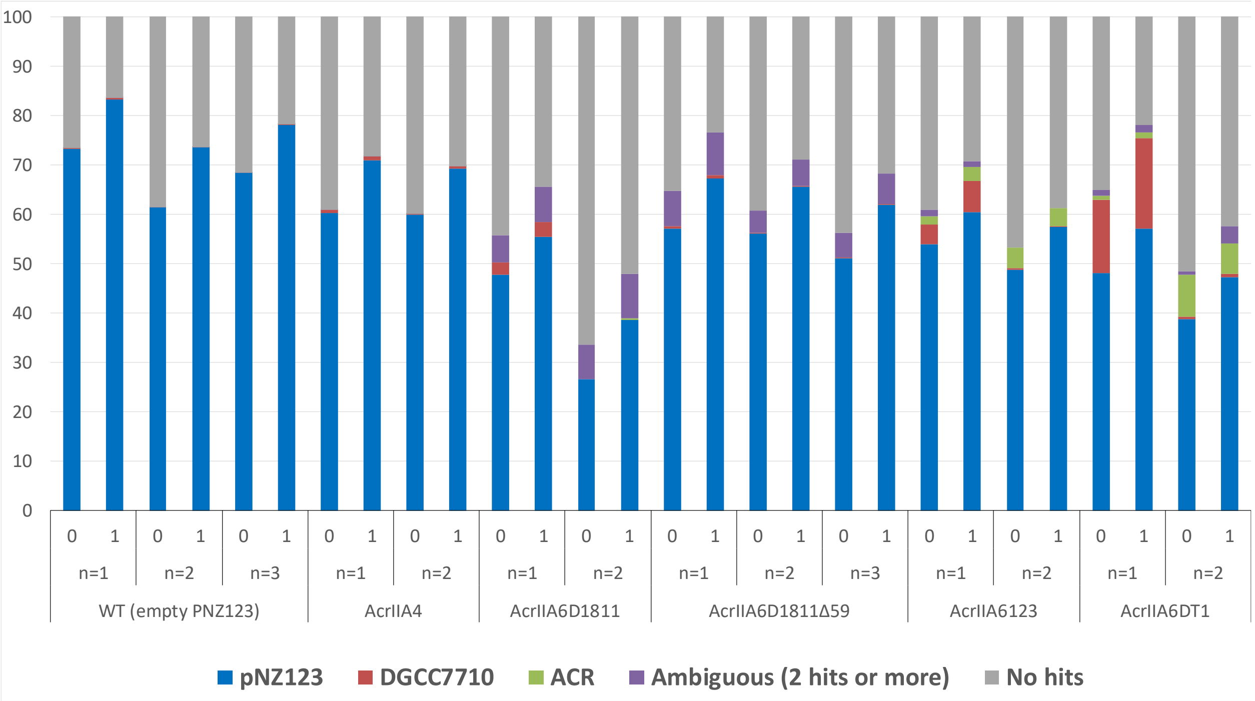

Figure S10. Relative abundance of spacer targets. Number of hits are provided for the various replicates, allowing 0 or 1 mismatch.

a

Spacer targets abundance

	Number of spacers	pNZ123	ACRs	DGCC 7710
-	261567	99.8%	0.0%	0.2%
AcrIIA4	49946	98.8%	0.0%	1.2%
AcrIIA5$_{D1126}$	804	87.3%	10.2%	2.5%
AcrIIA6$_{DT1}$	10012	75.5%	1.4%	22.7%
AcrIIA6$_{123}$	136	90.4%	2.9%	6.5%
AcrIIA6$_{D1811}$	1552	95.3%	0.0%	4.2%
AcrIIA6$_{D1811\Delta 59}$	6296	99.1%	0.0%	0.8%

b

Position of matching spacers on pNZ123

c

PAMs

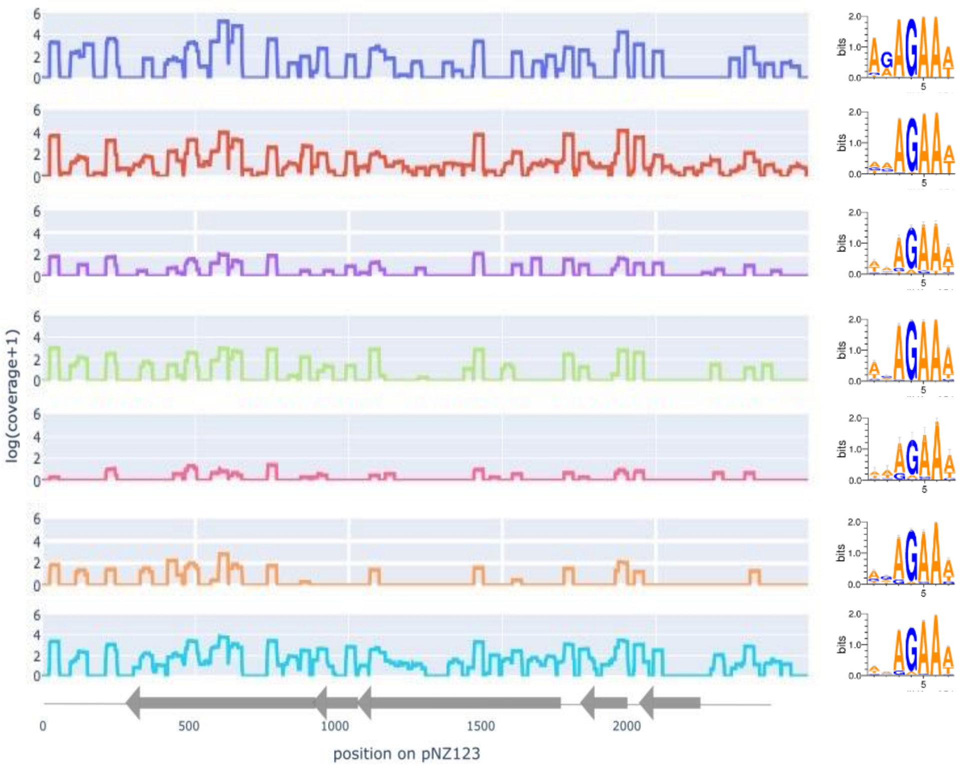

Figure S11. Newly acquired spacers primarily target the plasmid pNZ123 with no preference for a specific location. a) Relative abundance of spacers with a perfect match to targets on the plasmid, the *acr* gene, or the bacterial genome. b) Spacers targeting the plasmid pNZ123 were mapped but no patterns were detected for target locations. c) Seven nucleotides were extracted upstream from each spacer that targeted the plasmid sequence to identify the cognate PAMs for each condition and are represented using WebLogo.

Figure S12. Sequencing of PCR products of AcrIIA5

>WT_D1126

ATGGCATACGGAAAAAGCAGATACAATTCATATAGGAAGCGAAACTTCTCTATAAGCGACAATCAGCGTAGGGAATA
TGCTAAAAAAATGAAGGAGTTAGAACAAGCGTTTGAAAACCTTGACGGATGGTATCTATCTAGCATGAAAGATAGTG
CGTACAAAGATTTCGGAAAATACGAAATTCGCTTATCAAATCATTCAGCAGACAATAGATATCATGACCTAGAAAAT
GGTCGCTTAATCGTTAATGTTAAAGCTAGTAAATTGAACTTCGTTGATATCATCGAGAATAAACTTGGTAAAATCAT
TGAGAAGATTGATACTCTTGATTTAGATAAGTACAGATTCATTAATGCTACTAAATTGGAACGTGATATCAAATGCT
ACTATAAAGGCTATAAGACAAAGAAGGATGTAATCTAA

>ACR-Deletion1

ATGGCATACGGAAAAAGCAGATACAATTCATATAGGAAGCGAAACTTCTCTATGATATCATCGAGAATAAACTTGGT
AAAATCATTGAGAAGATTGATACTCTTGATTTAGATAAGTACAGATTCATTAATGCTACTAAATTGGAACGTGATAT
CAAATGCTACTATAAAGGCTATAAGACAAAGAAGGATGTAATCTAA

>ACR-Deletion2

ATGGCATACGGAAAAAGCAGATACAATTCATATAGGAAGCGAAACTTCTCTATAAGCGACAATCAGCATTGGAACGT
GATATCAAATGCTACTATAAAGGCTATAAGACAAAGAAGGATGTAATCTAA

Supplementary Table 1: Strains, phages, plasmids, and oligos used in this study

Strains	Description	Function	Source
NEB5α	*Escherichia coli*	Cloning	NEB
DGCC7710	*S. thermophilus*, host for phages 2972, D1126 and D3288	Sub-Cloning	1
SMQ-1335b	DGCC7710 w/CR1 spacer targeting 2972, D1811 and D3288	Sub-Cloning	2
SMQ-1339	DGCC7710 (pNZ123), CmR	Control for ACR activity	2
SMQ-1350	SMQ-1335b (pNZ123), CmR	Test for ACR activity	3
SMQ-1368	DGCC7710 (pNZAcrIIA4), CmR	Control for ACR activity	4
SMQ-1369	DGCC7710 (pNZAcr-1126), CmR	Control for ACR activity	4
SMQ-1370	DGCC7710 (pNZAcr-1811), CmR	Control for ACR activity	4
SMQ-1372	DGCC7710 (pNZAcr-DT1), CmR	Control for ACR activity	4
SMQ-1374	SMQ-1335b (pNZAcrIIA4), CmR	Test for ACR activity	4
SMQ-1376	SMQ-1335b (pNZAcr-1811), CmR	Test for ACR activity	4
SMQ-1378	SMQ-1335b (pNZAcr-DT1), CmR	Test for ACR activity	4
SMQ-1433	DGCC7710 (pNZAcr-123), CmR	Control for ACR activity	This study
SMQ-1434	DGCC7710 (pNZAcr-D1811-Δ59), CmR	Control for ACR activity	This study
SMQ-1435	SMQ-1335b (pNZAcr-123), CmR	Test for ACR activity	This study
SMQ-1436	SMQ-1335b (pNZAcr-D1811-Δ59), CmR	Test for ACR activity	This study
SMQ-1437	DGCC7710, w/CR1 spacer targeting the *acr* gene of D1126	Test for ACR activity	This study
SMQ-1438	DGCC7710, w/CR1 spacer targeting the *acr* gene of D3288	Test for ACR activity	This study
UY04	*S. thermophilus*, host for phage 123	Phage amplification	This study

Phages	Description	GenBank Accession	Source
123	Virulent *cos*-type phage<br>Host *S. thermophilus* UY04	MZ090946	This study
2972	Virulent *pac*-type phage,<br>Host *S. thermophilus* DGCC7710	AY699705	5
D1126	Virulent *pac*-type phage<br>Host *S. thermophilus* DGCC7710	N/A	4
D1126 ΔACR	Mutant of phage D1126 with a truncated ACR	MZ090947	This study
D3288	Virulent *pac*-type phage<br>Host *S. thermophilus* DGCC7710	N/A	4

Plasmids	Description	Function	Source
pNZ123	Vector, encodes chloramphenicol resistance	Negative control	3
pNZAcrIIA4	pNZ123 with *acrIIA4* inserted in the XbaI cut site	Expression of anti-CRISPR AcrIIA4	4
pNZAcrIIA5-D1126	pNZ123 with *acrIIA5*-D1126 inserted in the XbaI cut site	Expression of anti-CRISPR AcrIIA6D1126	4
pNZAcrIIA6-DT1	pNZ123 with DT1_g27 inserted in the XbaI cut site	Expression of anti-CRISPR AcrIIA6DT1	4
pNZAcrIIA6-D1811	pNZ123 with D1811_*g26* inserted in the XbaI cut site	Expression of anti-CRISPR AcrIIA6D1811	4
pNZAcrIIA6-123	pNZ123 with 123_*g24* inserted in the XbaI cut site	Expression of anti-CRISPR AcrIIA6-123	This study
pNZAcrIIA6-D1811-Δ59	pNZ123 with truncated D1811_*g26*	Expression of truncated anti-CRISPR AcrIIA6D1811	This study

Oligos	Sequence 5'-3'	Function	Source
Yc70	TGCTGAGACAACCTAGTCTCTC	CR1 locus screening (*St*)	6
RDS7rev	GGATCCGGATCCGTTGAGGCCTTGTTC	CR1 locus screening (DGCC7710)	6
dsCR1-F	TCGTCGGCAGCGTCAGATGTGTATAAGAGACAGTGCTGAGACAACCTAGTCTCTC	CR1 locus deep sequencing (DGCC7710)	This study
dsCR1-R	GTCTCGTGGGCTCGGAGATGTGTATAAGAGACAGGTTGTGCCAATAGCTCCTCG	CR1 locus deep sequencing (DGCC7710)	This study
CR3-fwd	CTGAGATTAATAGTGCGATTACG	CR3 locus screening	6
CR3-rev	GCTGGATATTCGTATAACATGTC	CR3 locus screening	6
pNZins_F	AATGTCACTAACCTGCCCCG	pNZ123 insert screening	2
pNZins_R	CATTGAACATGCTGAAGAGC	pNZ123 insert screening	2
123_ACRF	ATTACAGCTCCAGATCCAGTACTGAATTCTAACTCTCTCTTGCCCTGACT	pNZAcr123 construction	This study
123_ACRR	GAAAATATGCACTCGAGAAGCTTGAGCTCTCAACGTCAACAGTTTTCTTGA	pNZAcr123 construction	This study
pNZAcr D1811-Δ59 Fwd	ATGCTCGACTGCCTATTC	pNZAcr D1811-Δ59 inverse PCR	This study
pNZAcr D1811-Δ59 Rev	TTTGTTTCCTCCTTTAATTTATGC	pNZAcr D1811-Δ59 inverse PCR	This study
D1126_ACR_F	ATTACAGCTCCAGATCCAGTACTGAATTCTTCTGAAAAAGTTTGGGAAGTAGCT	Used to construct pNZAcr-1126, *acr* amplification, sequencing	4
D1126_ACR_R	GAAAATATGCACTCGAGAAGCTTGAGCTCTACTAACACCAGTTTGTCTTTCTAAAT	Used to construct pNZAcr-1126, *acr* amplification, sequencing	4
CR1-UY04-intern-R	GATGGTAGTGGTTTTTAATCC	CR1 locus screening (UY04)	This study
CR3-UY04-intern-R	CTTGGACTATCGAAACCC	CR3 locus screening (UY04)	This study
2972.19	CCGACGTTTCGAGTTTGGTC	Phage 123 sequencing	This study
DT104	CCGATGACATCATCACGGTT	Phage 123 sequencing	This study

Supplementary References

1. Barrangou, R. *et al.* CRISPR Provides acquired resistance against viruses in prokaryotes. *Science.* **315**, 1709–1712 (2007).
2. Hynes, A. P., Labrie, S. J. & Moineau, S. Programming native CRISPR arrays for the generation of targeted immunity. *mBio* **7**, 1–4 (2016).
3. Hynes, A. P. *et al.* An anti-CRISPR from a virulent streptococcal phage inhibits *Streptococcus pyogenes* Cas9. *Nat. Microbiol.* **2**, 1374–1380 (2017).
4. Hynes, A. P. *et al.* Widespread anti-CRISPR proteins in virulent bacteriophages inhibit a range of Cas9 proteins. *Nat. Commun.* **9**, 2929 (2018).
5. Lévesque, C. *et al.* Genomic organization and molecular analysis of virulent bacteriophage 2972 infecting an exopolysaccharide-producing *Streptococcus thermophilus* strain. *Appl. Environ. Microbiol.* **71**, 4057–4068 (2005).
6. Horvath, P. *et al.* Diversity, activity, and evolution of CRISPR loci in *Streptococcus thermophilus*. *J. Bacteriol.* **190**, 1401–1412 (2008).
